# Peripheral neuropathy and health-related quality of life in patients with primary Sjögren’s syndrome: a preliminary report

**DOI:** 10.1007/s00296-020-04543-2

**Published:** 2020-03-14

**Authors:** Marta Jaskólska, Magdalena Chylińska, Anna Masiak, Katarzyna Nowicka-Sauer, Mariusz Siemiński, Marcin Ziętkiewicz, Zenobia Czuszyńska, Zbigniew Zdrojewski

**Affiliations:** 1 Department of Internal Medicine, Connective Tissue Diseases and Geriatrics, Medical University of Gdansk, Gdańsk, Poland; 2 Department of Adult Neurology, Medical University of Gdansk, Gdańsk, Poland; 3 Department of Family Medicine, Medical University of Gdansk, Gdańsk, Poland

**Keywords:** Sjögren’s syndrome, Autoimmune neuropathy, Peripheral nervous system involvement, Health-related quality of life, Patient-reported outcomes

## Abstract

Sjögren’s syndrome (SS) is a chronic autoimmune disease with a wide spectrum of possible organ involvement. Peripheral (PNS) and central nervous system (CNS)-related symptoms may occur in the course of the disease. The aim of this study was to compare the health-related quality of life (HR-QOL) in SS patients with and without peripheral neuropathy. The study involved 50 patients with primary Sjögren’s syndrome (pSS). All patients underwent neurological clinical examination followed by nerve conduction studies (NCS) and rheumatological examination. Thirty-six-item Short Form Health Survey (SF-36) was used for evaluating HR-QOL. To assess pSS activity, the EULAR Sjögren’s Syndrome Disease Activity Index (ESSDAI) and EULAR Sjögren’s Syndrome Patient Reported Index (ESSPRI) were used. For the assessment of clinical disability due to peripheral neuropathy, the Overall Disability Sum Score scale (ODSS) was used. Additional evaluation of pain was performed with the use of the Visual Analogue Scale (VAS) and a semistructured interview. Twenty-three (46%) patients were diagnosed with peripheral neuropathy. The most common PNS manifestation was sensorimotor neuropathy (47%). Neurological symptoms preceded the diagnosis of pSS in eight patients. The following domains of the SF-36 form were significantly lower scored by patients with peripheral nervous system involvement: role-physical [0 (0–100) vs. 75 (0–100)], role-emotional [67 (0–100) vs. 100 (0–100)], vitality [40 (10–70) vs. 50 (20–75)], bodily pain [45 (10–75) vs. 55 (0–100)], and general health [20 (5–50) vs. 30 (0–50)] (*p* ≤ 0.05). Our study showed that peripheral neuropathy was a common organ-specific complication in SS patients. In pSS patients, coexisting neurological involvement with symptoms such as pain and physical disability may be responsible for diminished HR-QOL.

## Introduction

Sjögren’s syndrome (SS) is a chronic inflammatory disease with an autoimmune background that mainly affects middle-aged women (male to female ratio is 1:9). It is characterized by the presence of lymphocytic infiltrates in the exocrine glands (mainly salivary and lacrimal), which causes dryness of the eyes (xerophthalmia) and mouth (xerostomia). Sjögren’s syndrome may be primary (pSS)—it occurs then as an independent disease or secondary (sSS)—in the course of other systemic connective tissue diseases. Its prevalence is estimated at 0.1–1% of the population [1]. Systemic manifestations are common in pSS and play a major role in the prognosis. In the course of SS, neurological symptoms may affect both the peripheral (PNS) and central nervous system (CNS). Involvement of CNS was described in 6–48% of SS patients and PNS in 2–60% of patients [2, 3]. Involvement of the peripheral nervous system has a clinical picture of neuropathy and following subtypes were described: sensory neuropathies, axonal sensorimotor polyneuropathy, mononeuropathy, multiple mononeuropathy, demyelinating polyradiculoneuropathy, cranial neuropathy, and autonomic neuropathy [3]. Being a chronic progressive autoimmune disease, pSS may negatively affect physical, psychological, and social functioning [4, 5]. Therefore, pSS may potentially impair both the health-related quality of life (HR-QOL) and psychological condition. HR-QOL has been defined as “the degree to which one’s usual or expected physical, emotional, and social well-being are affected by a medical condition or its treatment” [5]. The fact that the patients’ awareness is growing and there is an increasing choice of therapeutic methods, puts physicians in need of a comprehensive assessment of patients, both disease activity and the patient-reported outcomes (PROs). Only in this way, we can count on good dialogue with the patient and compliance, which in turn will improve treatment outcomes. Routine use of patient-reported outcome measures (PROMs) will soon be unavoidable in both everyday clinical practice and clinical trials. Previous studies have demonstrated significant reduction of HR-QOL in pSS patients [5–7]. It was related to fatigue, pain, psychological distress, and sicca symptoms; however, the precise factors contributing to diminished HR-QOL in pSS remain unclear. To our knowledge, no study investigating the correlations between peripheral nervous system involvement and HR-QOL in pSS has been published. The aim of the present study was to compare the HR-QOL in pSS patients with and without peripheral neuropathy, and to assess the associations between neurological complications and various components of HR-QOL using the SF-36 Health Survey.

## Patients and methods

The study was approved by the Independent Bioethics Committee for Scientific Research of Medical University of Gdańsk, Poland (consent no. NKEBN/345/2011). All procedures were in accordance with the ethical standards of the 1964 Helsinki declaration and its later amendments. All patients gave their informed written consent.

### Patients

We studied a group of 50 unselected adult patients with the diagnosis of pSS who were under the care of the Department of Internal Diseases, Connective Tissue Diseases and Geriatrics of the Medical University of Gdańsk. Diagnosis of pSS was based on the 2002 American European Consensus Group criteria and Sjögren’s International Collaborative Clinical Alliance criteria published in 2012 [8, 9]. Exclusion criteria were: a diagnosis of other connective tissue disease, diabetes, and other conditions that may cause nerve damage, such as degenerative disc disease, alcohol or other toxic substance use, major organ failure, vitamin deficiencies, and other neurological conditions.

### Rheumatological assessment

For each patient, past and present systemic manifestations of pSS were recorded. Rheumatological assessment was carried out by one certified rheumatologist, and consisted of a detailed medical history, physical examination, pain evaluation, serological blood tests, assessment of disease activity, and functional outcomes.

### Neurological assessment and nerve conduction studies

All patients underwent clinical neurological examination followed by nerve conduction studies (NCS) of nine peripheral nerves (peroneal, tibial, sural, median-bilaterally, and ulnar-unilaterally). The NCS studies were performed following our standard laboratory methods in accordance with the recommended protocol of the American Association of Neuromuscular and Electrodiagnostic Medicine (AANEM) with the use of Medtronic’s Dantec Keypoint G4 [10]. Recordings were performed with skin temperature control (32°–34°). The lower limits of normal values were obtained based on a group of 50 age-matched healthy individuals. We used the European Standardized Telematic Tool to Evaluate Electrodiagnostic Methods (ESTEEM) guidelines to classify polyneuropathies as axonal, demyelinating, or mixed [11]. Both the clinical examination and NCS were carried out and evaluated by the same certified neurologist. Based on these results, patients were classified into two groups: with (PNS +) and without peripheral nervous system involvement (PNS −). A patient was classified as PNS + when at least following sings were found on clinical examination: sensory deficit (tactile of vibration) or paresthesia or neuropathic pain found in at least one anatomical area innervated by specific peripheral nerves or neural roots; flaccid paresis found in any of the limbs; reduction of at least two tendon reflexes in at least one limb; signs of demyelination or axonal injury found in NCS of at least two nerves, behind typical localization of entrapments. Involvement of cranial nerves was assessed by standard neurological examination. Presence of symptoms from any of the cranial nerves was interpreted as presence of neuropathy. Further comparative analyses were carried out between the two groups.

### Disease activity and functional outcomes

To assess SS activity, the EULAR Sjögren’s Syndrome Disease Activity Index (ESSDAI) was used [12]. The ESSDAI includes assessment of 12 domains: cutaneous, respiratory, renal, articular, muscular, PNS, CNS, haematological, glandular, constitutional, lymphadenopathic, and biological. Each domain is divided into 3–4 levels of activity with a score assigned. Moderately active disease is being defined as an ESSDAI ≥ 5.

To assess irreversible damage to organs, Sjögren’s Syndrome Disease Damage Index (SSDDI) was used [13]. SSDDI consists of damage scores of six organ groups: oral/salivary, ocular, neurologic, pleuropulmonary, renal impairment and lymphoproliferative disease. The higher the score, the greater the organ damage is present.

Additionally patients rated their disease activity using EULAR Sjögren’s Syndrome Patient Reported Index (ESSPRI) [14]. ESSPRI is a patient-administered questionnaire that evaluates dryness, fatigue, and pain, and each component is measured with a single 0–10 numerical scale. A score of 0 indicates the lowest disease activity.

For the assessment of clinical disability due to peripheral neuropathy, the Overall Disability Sum Score scale (ODSS) was used [15]. The ODSS evaluates the upper and lower limb functions. The score ranges from 0 to 5 in the upper limb and from 0 to 7 in the lower limb. A score of 0 indicates no limitations and the highest scores indicate maximum physical disability.

We additionally asked patients to evaluate the pain that they have experienced in the previous 2 weeks with the Visual Analogue Scale (VAS). Based on a semistructured interview, the pain was categorized into the following types: neuropathic, muscular, or articular. Neuropathic pain was characterized as numbness, pins, and needles sensations or burning sensation. Muscular pain was described as aching or stiffness of the limb and girdle muscles or a feeling that muscles have been overworked. Articular pain (arthralgia) was characterized as pain or stiffness limited to joints. We have decided to use our own questionnaire focusing on pain to get one simple and comprehensive tool for gathering qualitative and quantitative information about patient’s pain. As the patients were filling in the questionnaire under supervision of experienced neurologist or rheumatologist, all potential interpretation problems were solved, especially in terms of differentiating neuropathic from muscular pain.

### Evaluation of quality of life—SF-36

Thirty-six-item Short Form Health Survey (SF-36) is the most widely used instrument for evaluating HR-QOL in patients with rheumatic diseases [16, 17]. The Optum SF-36v2 Health Survey was used in the current study. It is a 36 question instrument for self-evaluation of several domains during the prior 4 weeks. It assesses eight domains: physical functioning (ten items), social functioning (two items), role-physical (four items), role-emotional (three items), mental health (five items), vitality (four items), bodily pain (two items), and general health (five items). Each domain evaluates health between 0 and 100 points, with 100 points indicating the best possible functioning [18]. The SF-36 is a global scale that has been useful in surveys of general and specific populations. It compares the relative burden resulting from various diseases and evaluates benefits resulting from different treatments [18].

### Statistical analysis

All statistical analyses were performed with Statistica version 13 (TIBCO Software Inc.). Normality distribution of the data was assessed with Shapiro–Wilk test. Continuous variables were compared using Mann–Whitney *U* test. Categorical variables were compared with the use of Chi-squared test. *p* value 0.05 or lower was considered significant.

## Results

Our group consisted of 50 patients with a median age 57.5 years (33–74), 48 (96%) were female, and two were male (4%). The median age at diagnosis of pSS was 53.5 years (23–69). Detailed clinical data are presented in Table 1. 40 patients (80%) reported subjective symptoms such as paresthesia or symptoms suggestive of neuropathic pain appearing periodically or permanently present. One patient who met the diagnostic criteria for peripheral neuropathy in NCS and neurological examination was asymptomatic. 23 of 50 (46%) patients met the criteria for the diagnosis of peripheral neuropathy and they were further referred to as PNS + patients. Patients without peripheral nervous system involvement (*n* = 27) were further referred to as PNS −. The most common PNS manifestation was sensorimotor neuropathy 11/23 (47%). Mononeuropathy was present in 6/23 (26%) patients, pure axonal sensory neuropathy in 1/23 (4.3%) patient, axonal motor neuropathy in 1/23 patient (4.3%), small-fiber neuropathy (SFN) in 1/23 (4.3%), and cranial nerve involvement was present in 4/23 (17.4%). One patient had both cranial and sensorimotor neuropathy. In one patient with normal NCS, the diagnosis of SFN was established on the basis of abnormal clinical and quantitative sensory testing (QST) findings performed in another laboratory.

**Table 1:** Clinical characteristics of studied sample and comparison between PNS + and PNS − groups

Characteristics	Studied sample *n* = 50	PNS + *n* = 23	PNS − *n* = 27	*p* value
Age [years, median (min–max)]	57.5 (33–74)	60 (33–74)	56 (34–68)	0.325
First symptoms [year of life, median (min–max)]	46 (18–66)	49 (18–64)	44 (23–66)	0.606
Diagnosis of pSS [year of life, median (min–max)]	53.5 (23–69)	56 (23–69)	51 (23–68)	0.402
Time to diagnosis [years, median (min–max)]	5 (0–26)	8 (0–20)	3 (0–26)	0.225
Disease duration [years, median (min–max)]	1.5 (1–15)	2 (1–10)	1 (1–15)	0.934
Symptoms; *n* (%)				
Xerophthalmia	49 (98)	22 (96)	27 (100)	0.273
Xerostomia	49 (98)	22 (96)	27 (100)	0.273
Parotid enlargement	29 (58)	17 (74)	12 (44)	0.035*
Oral mucositis	21 (42)	13 (57)	8 (30)	0.053
Positive Schirmer test	30 (60)	17 (74)	13 (48)	0.061
Vasculitis	11 (22)	7 (30)	4 (15)	0.183
Articular involvement	28 (56)	12 (52)	16 (59)	0.614
Gastrointestinal tract involvement	26 (52)	12 (52)	14 (52)	0.981
Cardiovascular system involvement	5 (10)	2 (9)	3 (11)	0.776
Respiratory tract involvement	25 (50)	15 (65)	10 (37)	0.047*
CNS involvement	11 (22)	7 (30)	4 (15)	0.183
Urinary tract involvement	25 (50)	12 (52)	13 (48)	0.776
Lymphadenopathy	19 (38)	14 (61)	5 (19)	0.002*
Lymphoma	2 (4)	2 (9)	0 (0)	0.073
Positive ANA	50 (100)	23 (100)	27 (100)	–
Positive anti-Ro/SS-A	35 (70)	16 (70)	19 (70)	0.979
Positive anti-La/SS-B	23 (46)	10 (43)	13 (48)	0.648
Rheumatoid factor	31(62)	13 (57)	18 (67)	0.665
Cryoglobulins	10 (20)	3 (13)	7 (26)	0.668
Hypocomplementemia	8 (16)	6 (26)	2 (7)	0.045*
Hypergammaglobulinemia	28 (56)	13 (57)	15 (56)	0.429
ESR > 30 mm/h	18 (36)	6 (26)	12 (44)	0.178
CRP > 5 mg/l	10 (20)	4 (17)	6 (22)	0.670
Treatment, *n* (%)				
Cyclophosphamide	4 (8)	4 (17)	0 (0)	0.010*
Steroids	37 (74)	19 (83)	18 (67)	0.194
ESSDAI [points; median (min–max)]	4 (0–21)	4 (0–21)	3 (0–15)	0.245
ESSPRI [points; median (min–max)]	5.165 (1.33–8)	6 (2.66–8)	5 (1.33–7.33)	0.187
SSDDI [points; median (min–max)]	4 (0–10)	4 (1–10)	3 (0–7)	0.055
ODSS [points; median (min–max)]	0 (0–7)	1 (0–7)	0 (0–4)	0.060

*PNS* *+* patients with peripheral neuropathy, *PNS* *−* patients without peripheral neuropathy, *pSS* primary Sjögren syndrome, *CNS* central nervous system, *ANA* antinuclear antibodies, *ESR* erythrocyte sedimentation rate, *CRP* C-reactive protein, *ESSDAI* EULAR Sjögren’s Syndrome Disease Activity Index, *ESSPRI* EULAR Sjögren’s Syndrome Patient Reported Index, *SSDDI* Sjögren’s Syndrome Disease Damage Index, *ODSS* Overall Disability Sum Score

*Statistically significant differences, *p* ≤ 0.05

Neurological symptoms preceded the diagnosis of pSS in 8 (35%) of 23 PNS + patients.

First, the two studied groups were compared in terms of clinical characteristics and results from laboratory tests. Details are presented in Table 1. The studied groups did not differ significantly in terms of age, duration of the disease (defined as number of years between the diagnosis and the moment of inclusion in the study), and time of symptoms onset as well as time from diagnosis. In comparison with PNS − in the PNS + group statistically significantly more prevalent were the following clinical manifestations: parotid enlargement (74% vs. 44%), respiratory tract involvement (65% vs. 37%), lymphadenopathy (61% vs. 19%), and hypocomplementemia (26% vs. 7%). In PNS + group, the use of cyclophosphamide, due to extraglandular manifestations, was statistically significantly more frequent (17% vs. 0%). There were no significant differences between PNS + and PNS − in terms of other organs involvement and results of ESSDAI, ESSPRI, SSDI, and ODSS. In PNS + group, the median ODSS score determining the severity of disability due to peripheral neuropathy was 1 (range 0–7).

Second, PNS + and PNS − groups were compared according to results obtained with the use of VAS-pain, the interview regarding type of pain and SF-36. The results are presented in Table 2. The median VAS-pain in PNS + compared to PNS − patients was 3 (range 0–7) and 0 (range 0–7), respectively (*p* = 0.229). Clinician’s assessment revealed that neuropathic type of pain was often observed in both subgroups. This type of pain was predominant in PNS + patients (16 of 23, 70%), while 11 of 27 PNS − patients (41%) suffered from neuropathic pain.

**Table 2:** Comparison of SF-36 and VAS scores between pSS patients with and without peripheral nervous system involvement

Domain	PNS + , *n* = 23 median (min–max)	PNS −, *n* = 27 median (min–max)	*p* value
Physical functioning	60 (0–90)	70 (20–100)	0.070
Social functioning	75 (25–100)	88 (0–100)	0.134
Role-physical	0 (0–100)	75 (0–100)	0.005*
Role-emotional	67 (0–100)	100 (0–100)	0.042*
Mental health	64 (20–96)	72 (32–100)	0.129
Vitality	40 (10–70)	50 (20–75)	0.040*
Bodily pain	45 (10–75)	55 (0–100)	0.038*
General health	20 (5–50)	30 (0–50)	0.044*
VAS-pain	3 (0–7)	0 (0–7)	0.229

*SF-36* Short Form Health Survey, *PNS +* patients with peripheral neuropathy, *PNS* *−* patients without peripheral neuropathy, *VAS* Visual Analogue Scale

*Statistically significant differences, *p* ≤ 0.05

In five domains of the SF-36 PNS + patients obtained significantly lower results than PNS − patients: role-physical [0 (0–100) vs. 75 (0–100)], role-emotional [67 (0–100) vs. 100 (0–100)], vitality [40 (10–70) vs. 50 (20–75)], bodily pain [45 (10–75) vs. 55 (0–100)], and general health [20 (5–50) vs. 30 (0–50)] (*p* ≤ 0.05).

## Discussion

Primary SS is a chronic disease with diverse clinical presentations which often leads to delayed diagnosis. Neurological complications and other extraglandular manifestations may precede the occurrence of typical symptoms of dryness (35% of patients in our study group). Delays in the diagnosis of pSS may contribute not only to the psychological distress, but also delay in starting the proper therapy and, therefore, may cause the development of systemic complications. Previous studies have shown large discrepancies in the prevalence and clinical picture of the nervous system involvement in pSS patients. Our study has clearly shown that peripheral nervous involvement is frequent, but remains under diagnosed in pSS patients, and it is correlated with worse HR-QOL. Patients with peripheral nervous system involvement obtained significantly lower scores in five domains of the SF-36: role-physical, role-emotional, vitality, bodily pain, and general health. There is an agreement in the literature that HR-QOL is impaired in pSS, but so far, it has been reported to be associated with fatigue, pain, sicca symptoms, and psychological distress [4, 18–20].

Fatigue (expressed as reduced vitality in SF-36) and pain are the most common extraglandular symptoms reported by patients suffering from rheumatologic diseases [21–23]. In population-based studies, the prevalence of persistent fatigue reaches approximately 20% among healthy individuals and up to 70% in patients with autoimmune diseases [21, 22]. The pathogenesis of persistent fatigue is still unknown. The proven impact of fatigue on patients’ quality of life is of great importance. Fatigue has been associated with mood disturbances, decreased motivation, impaired sleep, chronic pain, inflammation, and psychosocial variables, which makes it one of the most comprehensive medical issues [22, 23].

The two items of bodily pain domain assess the frequency of pain and its impact on daily functioning, but does not distinguish the type of pain and its intensity. We suggest that additional evaluation of the intensity of pain according to VAS scale and using a semistructured interview to try to categorize the type of pain into neuropathic, muscular, or articular is vital in these patients’ group. Even if the pain is in the range of mild-to-moderate like in our group, it should be emphasized that we are considering chronic pain accompanying the patient every single day. Moreover, one should not assume that every pain suffered by a patient with a rheumatic disease is articular. According to clinicians’ assessment, many of our patients suffered from neuropathic pain. It should be emphasized that physician should strive to determine the nature of the pain and thus to start appropriate treatment.

It is interesting to mention that in our study, there was a noticeable difference between pain scores on VAS scale and ESSPRI index, although the correlation between these two indicators was statistically significant (*r* = 0.53). The median score of pain on VAS in PNS + compared to PNS − patients was 3 (range 0–7) and 0 (range 0–7), respectively, while median score of pain on ESSPRI in PNS + compared to PNS − patients was 5 (0–8) and 4 (0–8), respectively. The possible scores on both scales range from 0 to 10, and in both, the same period of time was taken into account (2 weeks prior to the assessment). Previous surveys have indicated that ESSPRI correlates significantly with other patient-reported scales including VAS-pain. However, the ESSPRI index does not distinguish the type of pain and rating together with other components (fatigue and dryness) may cause an overstatement of the pain score. We hypothesize that more extensive pain assessment scale should be used in this group of patients to eliminate the negative effects of other symptoms on pain scoring and to distinguish the type of pain which is predominant to adjust the proper treatment.

Among the rheumatic diseases, functional disability most often affects patients with rheumatoid arthritis (RA) and systemic sclerosis (SSc). SS patients tend to be overlooked in this manner, because their disease is not “externally noticeable” like joint deformations in RA or contractures in SSc. However, concomitant neuropathy can significantly affect physical functioning in pSS patients. The median ODSS score determining the severity of disability due to peripheral neuropathy in our study was 1 (0–7) in PNS + group which reflects the worse every day functioning and may explain the low scores in role-physical domain. In PNS + patients, the neurological examination most often revealed sensory deficits and mild-to-moderate muscle weakness. However, in one patient, ataxic sensory neuropathy led to a significant disability due to severe loss of proprioception and kinesthesia.

When a difficulty in performing certain activity occurs, many of the patients adapt by starting to choose alternative ones. Also in many of them, psychological adaptation occurs, because expectations are being reduced [4]. This may be the explanation why the three domains physical functioning, social functioning, and mental health did not differ statistically between the subgroups.

These two factors, i.e., neuropathic pain and disability due to neuropathy, may be two important components that affect the HR-QOL. Further research is needed in this field.

Scores on SF-36 obtained from PNS + SS patients in our study are similar to those previously obtained from RA patients [24]. However, in RA patients, the low scores in physical domains resulted predominantly from joint involvement. Compared to systemic lupus erythematosus (SLE) patients, SS patients with neuropathy report poorer HR-QOL; they tend to score lower in physical functioning, mental health, bodily pain, and general health domains [25].

PNS + SS patients tended to score lower in physical functioning, role-physical, bodily pain, vitality, and general health domains compared to systemic sclerosis (SSc) patients [7]. In SSc patients, disease duration, gastrointestinal, and pulmonary involvement were negatively correlated with SF-36 physical domains, and the extent of skin involvement (measured with modified Rodnan skin score) was associated with reduced both physical and mental scores.

SS patients in general tended to have lower scores in vitality domain than patients with RA, SLE, and SSc [7]. This is intriguing, because SS has the best prognosis regarding life expectancy when compared with the above-mentioned diseases. This “low vitality” may result from nonspecific multiorgan symptoms, delay in the diagnosis, disease advancement at the time of diagnosis, and insufficient diagnosis of extraglandular complications. Also unrecognized nature of chronic pain and hence inappropriate treatment may be responsible.

Development of medicine with extensive diagnostic options and new treatment possibilities has improved mean life expectancies, leading to an increased number of patients with chronic diseases [26, 27]. Deterioration in physical and psychosocial functioning associated with chronic diseases has a negative impact on patients’ HR-QOL. This obliges healthcare professionals to take a comprehensive assessment of patients with consideration of both the disease activity and the patient-reported outcomes. Only this comprehensive approach could enable adjusting proper personalized treatment strategy working toward improving patients’ functional outcomes, psychological distress reduction, and improving HR-QOL.

Peripheral neuropathy in SS should be diagnosed and treated properly. Currently, there are no guidelines based on randomized trials regarding the treatment of patients with SS with nervous system involvement. Patients with oligosymptomatic and self-limiting course of the disease do not require pharmacological treatment. Immunosuppressive treatment is applied in case of high disease activity or progressive course of the nervous system involvement [28, 29]. In the symptomatic treatment of neuropathic pain, anti-epileptic drugs, antidepressants, and opioids are being used [28]. In the presence of pain, regardless of its nature and cause, effective treatment is necessary to improve patients’ QOL.

In summary, these preliminary results suggest a large unmet health burden. SS patients are a very diverse group of patients with possible involvement of every organ and system, and therefore, the rheumatologists’ broad knowledge and interdisciplinary approach to these patients are relevant. We should remember that patients value their QOL more than disease activity indexes or laboratory parameters. This is the first study that emphasizes the relationship between PNS involvement and worse HR-QOL in pSS patients. An attempt to expand the pain assessment with its categorization into muscular, articular, and neuropathic is also a novelty in this group of patients. The above conclusions indicate the direction of future research in this group of patients, focused on the rapid detection of organ-specific complications with subsequent appropriate treatment. In addition, they indicate the need to pay more attention to the subjective symptoms reported by patients and their careful assessment. Further research seems to be needed on the pathomechanism of pain formation and its perception in pSS. Also the development of new scales to accurately assess pain and other patient-reported complaints seems necessary. It should be emphasized that pain in rheumatological patients is a much more complex problem than it seemed. As an attending physician, rheumatologist should take care of every aspect of the patient’s life, starting from controlling disease activity, effective pain treatment, through improvement of daily functioning, and finally mental well-being.

## Conclusion

Our study showed that peripheral nervous involvement is frequent in pSS patients and it is correlated with worse HR-QOL. We hypothesize that in pSS patients coexisting neurological complications with symptoms such as pain and physical disability, may be the reason for diminished HR-QOL. To get the complete picture of SS patients, a holistic approach starting with a comprehensive assessment of disease activity and PROs is needed. Combining personalized therapies, including immunosuppression, symptomatic pain management, physiotherapy, and psychological support, can improve compliance and treatment outcomes and patients’ HR-QOL.

### Limitations

The study was conducted in a tertiary care university hospital, and thus, referral bias may have played a role in patient selection. Additionally, we did not have the possibility to conduct neurophysiological testing in cases where a pure small-fiber neuropathy was suspected.

One of the weaknesses of our study was lack of unambiguous differentiation between SS-related neuropathy and other forms of neuropathy (e.g., chronic inflammatory demyelinating neuropathy). To diagnose a neuropathy related to a disease, one should prove existence of a relation between the presence, course, and therapy of the disease, and the presence and course of neuropathy. In case of SS establishing direct link between the autoimmunological disease and a polyneuropathy (diagnosing “SS-related” polyneuropathy) would take years of observation. On the other hand, the aim of this study was to assess the association between involvement of peripheral nervous system and the quality of life of subjects with SS. For such an aim, the exact etiology of polyneuropathy is not decisive.

The main advantage is the fact that study is a cross-sectional one with a thorough neurological and rheumatological assessment performed by one certified specialist. Much of the research in this topic was based on retrospective assessment. Additionally, only patients with pSS were included in the study. Patients with additional connective tissue diseases as well as patients with diabetes, degenerative disc disease, and other neurological conditions were excluded from the study.
